# Poland's syndrome

**DOI:** 10.11604/pamj.2015.21.294.7599

**Published:** 2015-08-24

**Authors:** Samia Frioui, Faycel Khachnaoui

**Affiliations:** 1 Service de Médecine Physique et de Réadaptation Fonctionnelle, CHU Sahloul, Faculté de Médecine “Ibn El Jazzar”, Sousse, Tunisie

**Keywords:** Poland′s Syndrome, adolescent, pectoralis major muscle

## Image in medicine

Poland's syndrome is characterized by agenesis of the pectoralis major muscle and mammary agenesis associated to the presence or not of bone or renal alterations. In rare cases, severely affected individuals have abnormalities of internal organs such as a lung or a kidney, or the heart is abnormally located in the right side of the chest (dextrocardia). It has been estimated to occur in 1 to 3 per 100,000 newborns. In Poland's syndrome, males predominate by at least 3:1 and usually the right side is affected in 75% of cases. The cause of Poland's syndrome is unknown. Researchers have suggested that it may result from a disruption of blood flow during development before birth. We report a case of an adolescent patient who consulted for back pain. Physical examination showed left anterior chest-wall depression, lateral to the nipple. There were no other anomalies seen on further systemic evaluation. Neurological examination and skeletal survey were normal. Poland's syndrome was diagnosed with absence of left pectoralis major muscle and mammary agenesis. A global evaluation of the patient was carried out with no findings of other markers of the disease. Mild cases of Poland syndrome without hand involvement may not be evident until puberty, when the differences between the two sides of the chest become more apparent. During adolescence, the disease assumes a risk factor due to alteration of the patient's body image and consequent reduction in self-esteem.

**Figure 1:**
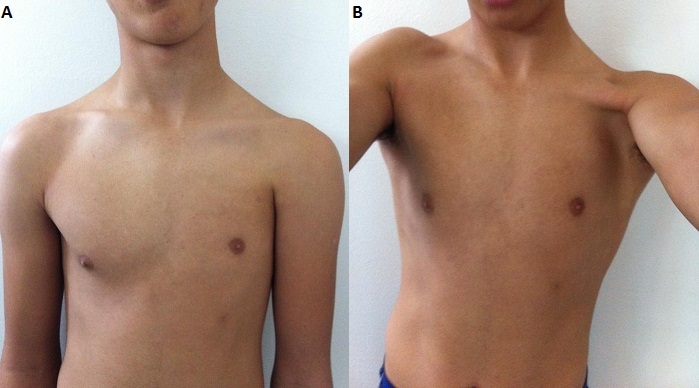
the appearance of the patient with Poland's syndrome. A: absence of the left pectoral muscles, superior localized left small nipple-areola complex; B: left anterior chest-wall depression and flatness of the left pectoral region

